# Supplementary material for: In vivo Validation of Bimolecular Fluorescence Complementation (BiFC) to Investigate Aggregate Formation in Amyotrophic Lateral Sclerosis (ALS)
Source: Mol Neurobiol. 2021 Jan 7;58(5):2061–74. doi: 10.1007/s12035-020-02238-0 (PMC8018926; doi:10.1007/s12035-020-02238-0)
Supplement: Supplementary file 1 — (DOCX 1129 kb) [file 12035_2020_2238_MOESM1_ESM.docx]

***In Vivo* Validation of Bimolecular Fluorescence Complementation (BiFC) to Investigate Aggregate Formation in Amyotrophic Lateral Sclerosis (ALS)**

Emily K Don^a^, Alina Maschirow^a^, Rowan A W Radford^a^, Natalie M Scherer^a^, Andres Vidal-Itriago^a^, Alison Hogan^a^, Isabel Formella^a^, Jack J Stoddart^a^, Thomas E Hall^b^, Albert Lee^a^, Bingyang Shi^a^, Nicholas J Cole^a^, Angela S Laird^a^, Andrew P Badrock^a^, Roger S Chung^a^, Marco Morsch^a^

^a^ Centre for Motor Neuron Disease Research, Faculty of Health and Medical Sciences, Department of Biomedical Science, Macquarie University, Sydney, NSW 2019, Australia.

^b^ Institute for Molecular Bioscience, The University of Queensland, QLD 4072, Australia.

**Corresponding authors:**

Macro Morsch marco.morsch@mq.edu.au

Roger Chung roger.chung@mq.edu.au
Andrew Badrock andrew.badrock@manchester.ac.uk

**
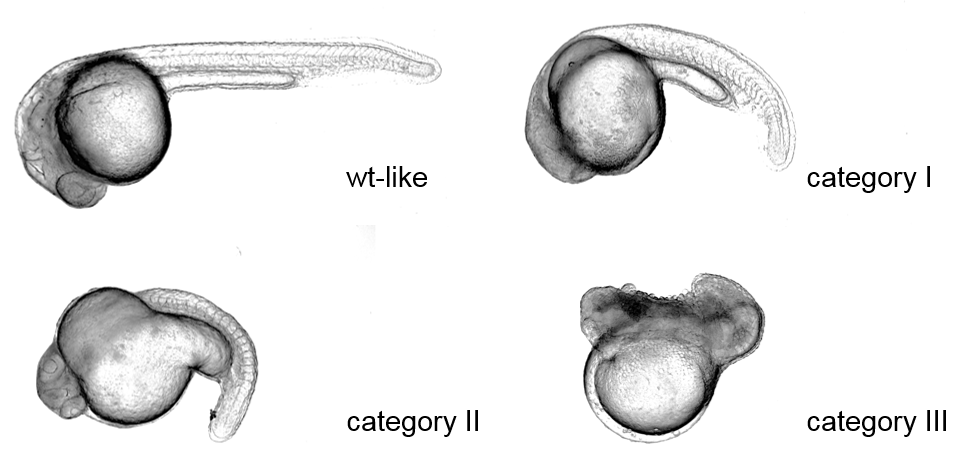

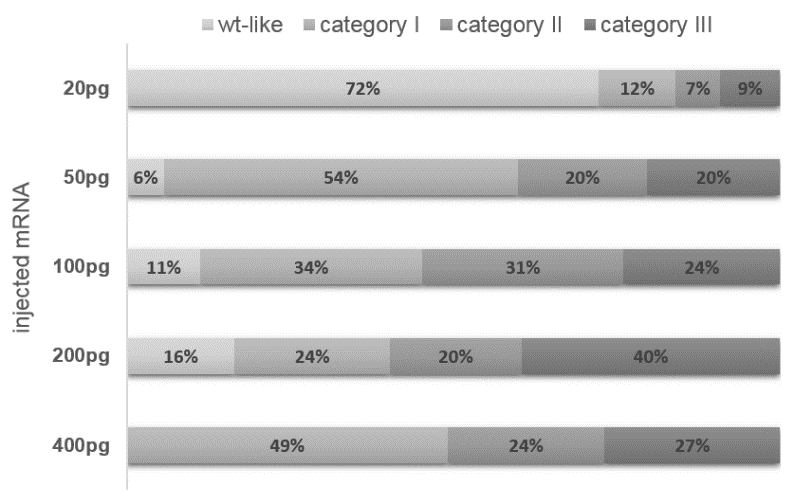
**

**a**

**b**

**c**

**d**

**
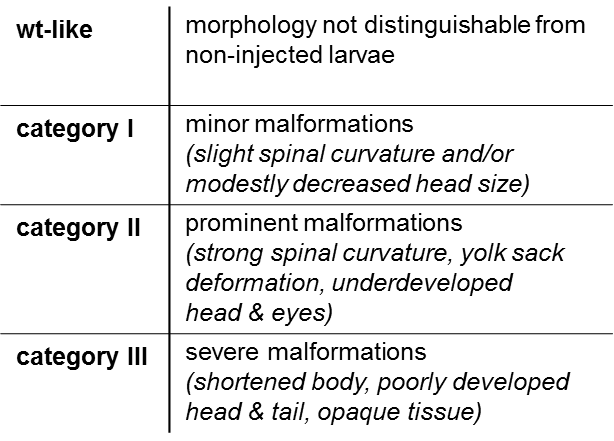
**

**
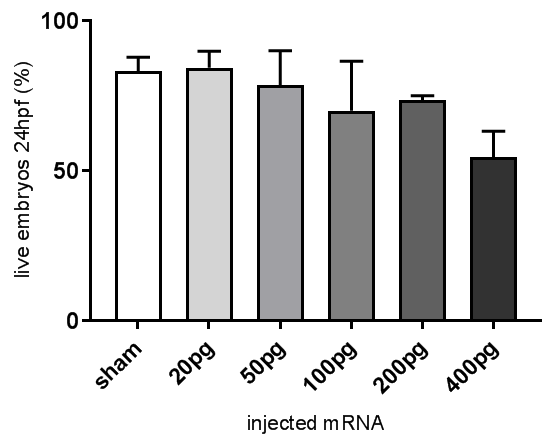
**

**Supplemental Fig 1 Effect of TDP-43 BiFC mRNA microinjections on zebrafish survival and morphology**

**a**: Zygotes at one-cell stage were microinjected with different amounts of wtTDP-43 BiFC mRNA and screened for survival at 24 hpf. Doses refer to the total of both complementary mRNAs. Results are shown as percentage of non-injected control embryos, and data are pooled from 3 independent experiments. **b**: Representative pictures and classification of morphological abnormalities observed at 24 hpf after wtTDP-43 BiFC mRNA microinjections. **c**: Description of morphological criteria used to categorize wtTDP-43 BiFC mRNA microinjected embryos. **d**: Quantitative evaluation of morphological phenomena in wtTDP-43 BiFC mRNA microinjected embryos at 24hpf, based on data from 3 independent experiments with a minimum of n=20 for each dose.


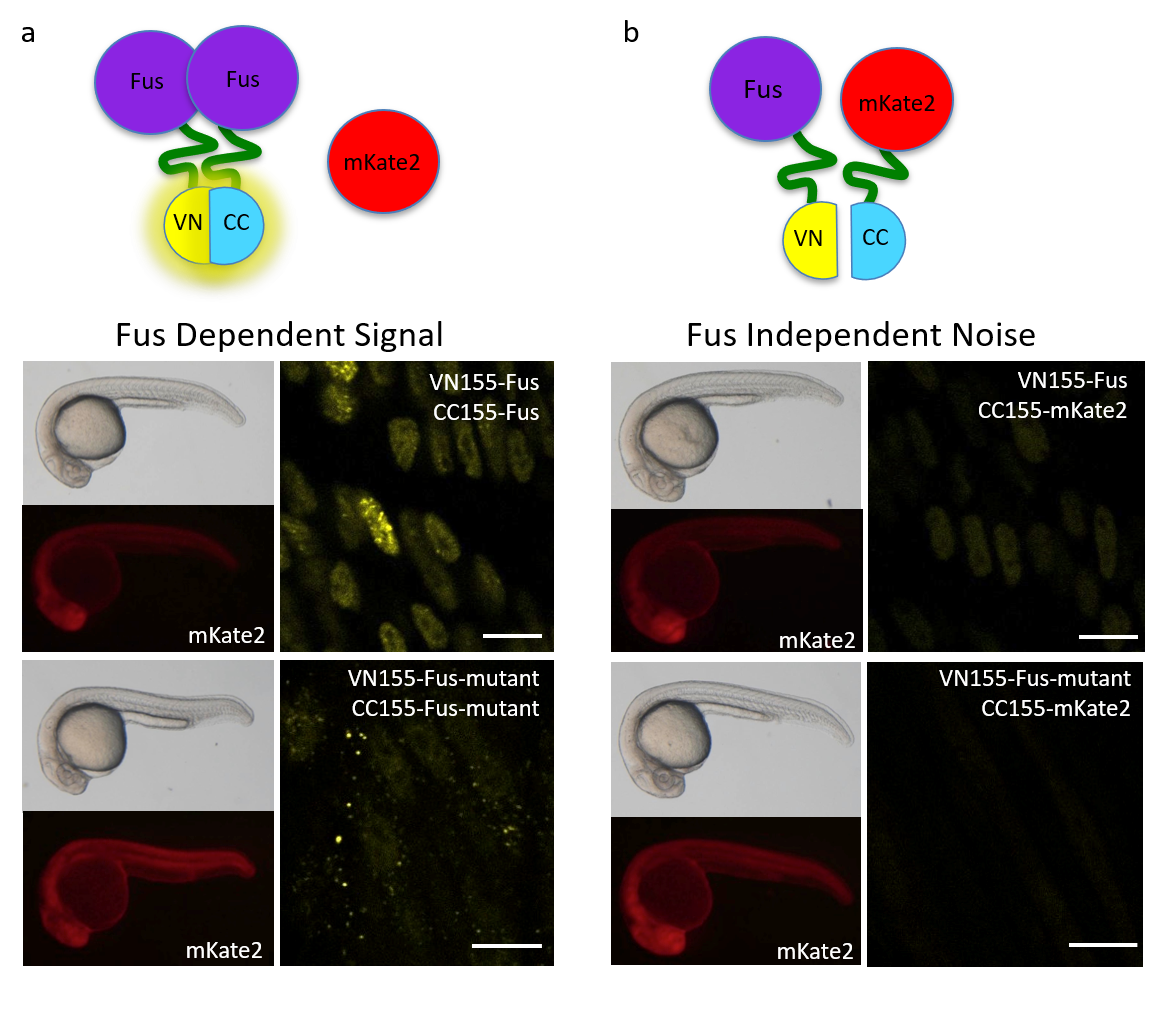


**Supplemental Fig 2 Fus aggregation in zebrafish is specific**

Fus-BiFC complementation assays. **a:** Fus dependent signal: 400 pg H2B-mCerulean3, 100 pg VN155-Fus (VN155-Fus-mutant), 100 pg CC155-Fus (CC155-Fus-mutant) and 40 pg mKate2 mRNA were co-injected into 1-cell stage wildtype embryos. Representative pictures of the zebrafish wild-type Fus (top panel) and mutant Fus (bottom panel) BiFC signal at 28 hpf in the somites over the yolk extension. Nuclear or cytoplasmic Fus-mVenus signal is detected in Fus dependent assays. High-magnification images also used in Figure 4. **b:** Fus independent noise: 400 pg H2B-mCerulean3, 100 pg of VN155-Fus (or VN155-Fus-mutant) and 50 pg of CC155-mKate2 mRNA were co- injected into 1-cell stage wildtype embryos. Representative pictures of the zebrafish wild-type Fus/mKate2 (top panel) and mutant Fus/mKate2 (bottom panel) BiFC signal at 28 hpf in the somites over the yolk extension. Scale bars represent 10 µm.
